# How Does Higher Education Influence Attitudes Towards Muslims? Examining Mechanisms That Reduce Prejudice Within UK Universities

**DOI:** 10.1111/1468-4446.70113

**Published:** 2026-04-07

**Authors:** Tom Fryer, Mathew Guest, Kristin Aune, Lucy Peacock

**Affiliations:** ^1^ Manchester Institute of Education University of Manchester Manchester UK; ^2^ Department of Theology and Religion Durham University Durham UK; ^3^ Department of Criminology, Social and Social Policy Loughborough University Loughborough UK; ^4^ Department of Sociology University of Warwick Coventry UK; ^5^ The Woolf Institute University of Cambridge Cambridge UK

**Keywords:** anti‐Muslim prejudice, higher education, political attitudes, religion, religious prejudice, universities

## Abstract

This article examines the relationship between encounters with religious diversity and the perspectives people form about Muslims. Its empirical focus is individuals studying at UK universities. Previous research suggests Muslims are amongst those most subject to negative prejudice in the UK, this being structured around racial or ethnic prejudice and negative judgements about Islam as a religion. Universities, on the other hand, generally retain a reputation for upholding values of equality, diversity and inclusion. While this reputation is not uncontested, evidence suggests high proportions of students affirm positive views about minority groups, including Muslims. This article examines longitudinal data from a national survey of university students to clarify how they view Muslims and examine how these views might have been shaped by their experience of university. Focusing on students' encounters with religious diversity, the article examines different mechanisms within the university experience that appear most significant in shaping a more positive outlook on Muslims. In so doing, it assesses the capacity of universities to drive progressive attitudinal change and investigates the social mechanisms that bring this about, highlighting in particular the initiation of ‘proactive encounters’ with other students affirming different worldviews.

## 1 Introduction

Despite a long history of post‐migration settlement, and being the nation's largest religious minority, Muslims remain a persistent focus of opposition and hostility within the United Kingdom. Heightened public visibility of Muslims as a religious minority during the late 1980s and 90s—especially following the Rushdie Affair (Modood 1990) ‐ precipitated widespread antagonism, leading the Runnymede Trust to identify a distinctive form of prejudice, Islamophobia (Runnymede Trust 1997). Its persistence was noted in 2018, by the UK's All‐Party Parliamentary Group on British Muslims, highlighting the discrimination and prejudice Muslims face ‘in employment, housing, education, the criminal justice system, social and publick life and political or media discourse’ (APPG on British Muslims 2018, 9). Academic studies have revealed how anti‐Muslim prejudice has become embedded within the institutional life of British schools (Holmwood and O'Toole 2018), universities (Saeed 2019; Scott‐Baumann et al. 2020), and local government (O'Toole et al. 2016). Such trends appear to have been heightened following terrorist attacks carried out in the name of Islam on UK soil, reinforcing a perception of Muslims as a security threat (Hussain and Bagguley 2012). This notion has fed into the government's counter terrorism Prevent strategy, which imposes safeguarding guidelines on publick bodies—including universities (Spiller et al. 2018)—that, some argue, reinforce anti‐Muslim prejudice (Brown and Saeed 2015; Scott‐Baumann et al. 2020).

In previous decades, prejudice towards Muslims is largely understood to have been conflated with a more generalised racism, with Muslim migrants from Pakistan treated similarly to Hindu and Sikh migrants originating from India, or indeed Christians from the Caribbean (Modood 2019, 48). Related tendencies have been exposed more recently by the campaign to exit the European Union (Brexit) in 2016, and the far‐right riots across the UK in 2024. Hostility towards Muslims is associated with nativist perspectives on British identity, even when Muslim migration to Britain has been occurring for well over 100 years (Ansari 2004), the majority of Muslim citizens are now UK‐born, and the vast majority view themselves as British (MCB 2024). But this ‘ethnocentric’ tendency is only partially driven by racially‐inflected ideas of who was born in Britain and who has ‘British heritage’; it is also bound up in perceptions about who upholds ‘British customs and traditions’ (Sobolewska and Ford 2020, 62), and hostility towards Muslims is often justified in these terms.

Within this broader context, the potential role of UK universities in challenging anti‐Muslim prejudice is especially significant, for several reasons. First, and despite persistent ‘myths’ about the ‘traditional student’ that overlook minority experiences (Sykes 2021), universities often exhibit more cultural and religious diversity than students experience in ‘home’ environments, providing a useful test case for how exposure to identity difference shapes attitudes towards Muslims as the UK's largest religious minority. Second, to consider how universities influence perspectives on Muslims is to probe one of the most deeply embedded assumptions about higher education (HE) in western capitalist democracies, that is that universities broaden the mind and hence foster positive orientations towards forms of diversity, whether expressed in terms of gender, sexuality, ethnicity and race, or religion (Delanty 2001). Finally, to interrogate the capacity of universities to contest anti‐Muslim prejudice is to shed light on the social mechanisms best placed to tackle racial and religious prejudice more generally. It is one thing to trace patterns of attitudinal change, quite another to identify the processes whereby such changes are brought about.

The present article draws on new longitudinal data from a national survey of students to investigate two questions (a) how do students' views of Muslims change during their time at university and (b) what is the role of the university experience in influencing this change?

## 2 Literature Review

### 2.1 Attitudes to Muslims in the UK

Attitudinal data from UK citizens suggest a persistent wariness towards Muslims, albeit one that is variable over time, and by class and political persuasion (Jones and Unsworth 2024). Demographer Clive Field reviewed 64 opinion polls during the 2007–10 period, estimating that up to two fifths of British people believe Muslims ‘do not want to fit into British society’ and ‘have little to offer Britain’ (Field 2012, 158). Some evidence suggests the numbers sharing such views have subsequently declined, with 17%, according to the 2018 British Social Attitudes (BSA) Survey, describing their perspective as ‘very’ or ‘somewhat negative’. Further decline was evidenced by Addario et al. (2024), who analysed BSA data from 2022, generating sub‐classes defined by attitudes towards different minorities. One, labelled ‘Wary of Muslims’, made up 11% of respondents, characterised by a tendency to be ‘fairly neutral towards the impact of immigration, likely to be comfortable with Black people, but uncomfortable with and negative towards Muslims and to a lesser extent immigrants’ (Addario et al. 2024, 8). The analysis underlines how hostility towards Muslims, while apparently declining and exhibiting some commonalities with other forms of racism, constitutes a special case whose ideological focus could lend it resilience.

This possibility is reinforced by Jones and Unsworth (2024), who draw on 2021 national survey data to argue that Islamophobia in Britain takes both racially and religiously oriented forms, and that these are overlapping but distinct. The first focuses on prejudice towards Muslims as people, the second on what are assumed to be Islamic beliefs (e.g., a literalist approach to religious texts). The authors find that, when asked about different categories of people, more respondents (25.9%) express negative feelings towards Muslims than any other group, apart from Gypsy and Irish Travellers. The figure is 8.5% for Jews and 6.4% for black people (2024, 10). According to the same study, the strongest drivers of anti‐Muslim prejudice are gender (being male) and voting behaviour (e.g., voting Conservative in the 2019 general election). They also find evidence that social class plays an important role, with working class and middle‐class respondents maintaining significant levels of anti‐Muslim sentiment, but with each suggestive of a different pattern. Using regression models, Jones and Unsworth find that ‘working‐class opposition to Muslim migration dissolves into racialised anti‐immigration sentiment, but middle‐class animus towards Islam focuses on Islam’ (2024, 17). The latter the authors link to misplaced middle‐class confidence in making judgements about Islam, and a pattern evidenced in other studies (e.g., Jones et al. 2019) that suggests anti‐Islam feelings function as a respectful form of prejudice in 21st century Britain, including among politically liberal citizens (cf. C. Allen 2013). As the authors argue, by contrast with racialised forms of anti‐Islam prejudice,

‘Scriptural determinist’ narratives about Islam…were more widespread, occurring also among those from middle‐class socioeconomic groups and with liberal political leanings. These narratives were also offered unselfconsciously: they were not seen by participants as potentially prejudiced—as something that would attract social censure—but as obvious common sense.

(Jones and Unsworth 2024, 18)

In other words, there is a tendency to view opposition to Islam as acceptable because Islam as a religious tradition is assumed to embody especially objectionable beliefs.

### 2.2 Attitudes to Muslims Among University Students

Notwithstanding the subtlety reported by Jones and Unsworth, levels of anti‐Muslim prejudice appear to be lower among younger generations and among the middle classes. Taking these demographic forces alongside the reputation of universities for upholding principles of equality, diversity and inclusion, we might be unsurprised to find students are less inclined towards anti‐Muslim prejudice than the general population. According to the 2018 BSA survey, 17% of the general population described their perspective on Muslims as ‘very’ or ‘somewhat negative’. That number drops to 13% among young adults, and a 2017 survey by Guest et al. (2020) suggests that university students are even less suspicious, with only around 6.5% affirming a comprehensively negative view. The same survey suggests over 70% of students at UK universities believe Muslims have made a ‘valuable contribution to British life’ (fewer than 10% disagree). Drawing on the same national data cited above, Field (2012) suggests up to three quarters of Britons claim limited or no knowledge of Islam; Guest et al.’s figure for university students is just below 60%. Students have a much more positive perspective on Islam and Muslims, even when a large proportion acknowledge their limited knowledge, which could suggest the distinct profile of university students has more to do with ethos and campus culture than it does with formal learning. Nevertheless, the data analysed by Guest et al. (2020) mirrors the pattern found by Jones and Unsworth (2024), that is respondents were much more likely to affirm negative views about Islam as a religious tradition—especially when associated with conservative teachings about other religions or the status of women—than about Muslims as people.

Prior studies of HE and students' attitudes tend to agree on a liberalising tendency. Individuals who have graduated from university exhibit attitudes that are generally more positive about minorities and migrants than those who have not had a university education. The evidence in favour of this association over time and across western cultural contexts is fairly strong (Borgonovi 2012; Weakliem 2002); however, the precise character of this link remains under‐researched. Indeed, some studies suggest this variable may have been overstated, with attitudinal tendencies attributable not to HE, but to the demographics of university recruitment—those who go to university being predisposed to adopt liberal values due to other factors, like early socialisation—or of post‐university life experience, such as not having to compete with migrants for lower paid employment (Persson 2015; Simon 2022; Fryer 2023).

Within those studies that have explored the mechanisms by which HE might shape students' attitudes, common assumptions point to a combination of critical thinking taught in universities, encounters with cultural diversity common in universities, and a general ethos of inclusivity associated with the university sector. Such assumptions have been subjected to significant critical scrutiny in recent scholarship, especially in light of the apparently performative, rather than substantial, commitment of universities to diversity agendas (Ahmed 2012), and the ways in which universities incubate or sustain patterns of prejudice found elsewhere in society (Arday and Mirza 2018; Law et al. 2004; Scott‐Baumann et al. 2020). Other mechanisms have been explored by other studies, from the content of curricula within different academic disciplines (Abbas et al. 2016; Muddiman 2020) to the social encounters and relationships distinctive to university contexts (Crossley and Ibrahim 2012; Dey 1996; Valentine et al. 2010; Yılmaz 2024). While taking the debate further, this body of research has not, thus far, drawn on large‐scale longitudinal data to trace patterns of attitudinal change among students at a national level, and hence probe directly the capacity of universities as agents of inclusivity across the UK HE sector. Our own research responds to this gap, while also addressing the distinctive ways in which encounters with religious difference at university contribute to the formation of perspectives on Muslims.

### 2.3 Religious Dimensions of University‐Based Attitudinal Change

Research on higher education in the UK ‐ with a few exceptions (e.g., Guest et al. 2013; Hopkins 2011; Peacock et al. 2023; Scott‐Baumann et al. 2020)—lacks a serious consideration of factors relevant to religious identities, such as students' encounters with religious diversity. This is perhaps unsurprising given the secularisation of UK institutions and comparatively low levels of religious participation within the wider population (Curtice et al. 2019). But it hampers sociological attempts to understand prejudice that is religiously oriented. Here, we need to turn to the US literature, which responds to a HE sector in which religion plays a far more salient role, whether via the institutional identities of Christian colleges, or in contending with the needs of a more religious student population (Hill 2017). In the US, a substantial body of research has emerged from the fields of sociology and education, addressing religion as an explanatory variable—an institutional or individual‐level factor in shaping attitudinal and educational outcomes (e.g., Small and Bowman 2011)—and as an outcome variable, examining how the religious identities of students are reinforced, challenged or reconfigured by their experiences of HE (e.g., Glanzer et al. 2014; Reimer 2010; Mayrl and Uecker 2011).

The US scholarship has also examined the specific case of attitudes towards Muslims (e.g., Cole et al. 2020; Rockenbach et al. 2017). Shaheen et al. (2023) measured changes in attitudes towards Muslims among non‐Muslim students in their first year of college, using longitudinal survey data to capture degrees and drivers of change. They conclude that increased appreciative attitudes towards Muslims are associated with campus culture (including institutional commitment to the appreciation of Muslims and the prevalence of religion, spirituality or interfaith programmes on campus), the provision of spaces for the support and expression of students' religious identities, whether non‐Muslim students have forged friendships with Muslims at college, and exposure to interfaith activities. A further important factor is what the authors call ‘provocative encounters’, that is ‘a critical moment in which students reevaluate assumptions about their own and others’ worldviews as a result of challenging discussions, disagreements, and even criticism…’ (Shaheen et al. 2023, 198).

The present analysis responds to the literature reviewed above in three respects. First, in substantive terms, it asks about a specific topic highly relevant to political and value divisions within contemporary Britain, that is the status of Muslims. We build on Fryer's (2023) longitudinal analysis of British Election Study data, which found a small increase in liberal views among university students during their period of study. Notably, of the two measures adopted, it was a shift towards a more pro‐immigration stance, rather than a shift to the left (in traditional political terms), that was more prominent. Insofar as this may indicate a particular subset of values especially prone to revision during university—related to religious and cultural difference as encountered by young adults—our analysis probes this possibility further. Second, this article investigates the specific drivers of attitudinal change by treating experiential dynamics—encounters with religious difference—as explanatory variables. In other words, rather than taking level of education as the key, generic driver, we focus on dimensions of the university experience that are directly relevant to our research questions. Specifically, we focus on measures of encounter with religious diversity *during* university in order to assess the significance of on‐campus diversity, and engagement with religious diversity *before* university, in order to distinguish variables specific to the university experience. Third, we go beyond passive encounters with religious diversity and consider active engagement with religious difference. Inspired by Shaheen et al. (2023) concept of ‘provocative encounters’, we set out to explore ‘proactive encounters’, in which students *initiate* a conversation with someone of a different religious or non‐religious perspective to learn more about their beliefs and values. Unlike the concept of ‘provocative encounters’ applied in US studies (e.g., Mayhew et al. 2023), our application of ‘proactive encounters’ does not assume students consequently reevaluate worldview assumptions. Instead, we separate the encounter from its consequences, in order to test, specifically, the impact of proactive encounters on attitudes towards Muslims.

## 3 Methodology

This article analysed longitudinal student survey data (*n* = 996) from The Interfaith Diversity Experiences and Attitudes Longitudinal Survey (IDEALS UK), which adapted questions from a US‐based project (Rockenbach et al. 2020) to a UK context. The data collection involved two surveys in Autumn 2021 (*n* = 4401) and Autumn 2022 (*n* = 4618), using a quota sampling method applied to an existing panel of UK‐based young adults by survey provider YouthSight.^1^ The longitudinal sample consisted of undergraduate (*n* = 890) and master's (*n* = 106) students from 116 UK universities, and contains some bias, for example overrepresentation of women, undergraduates, full‐time programmes, more prestigious HE institutions (HEIs), and STEM subjects (see Supporting Information S1: Table A1). Hence, while the sample captures responses from across the UK HE sector, and therefore provides evidence about various diverse student groups and their attitudes, the article's conclusions do not claim to be wholly representative of the overall UK HE population.^2^ For example, it is possible the overrepresentation of more prestigious HEIs in our sample, which tend to be less socially diverse (Boeren 2024), may influence students' experiences. Ethical approval was received from Coventry University (Approval Number: P125182).

To assess students' views of Muslims, and how these changed over one calendar year, we investigated responses to the question ‘*To what extent do you agree with the statement: In general, Muslim people make positive contributions to society*’. This item was chosen based on similarities to previous surveys (e.g., Guest et al. 2020), and building on Jones and Unsworth (2024), by not prioritising racialised anti‐Islam sentiments, the item may better capture more subtle forms of anti‐Muslim prejudice. This item was answered on a Likert scale: Strongly Disagree; Disagree; Neither Agree nor Disagree; Agree; Strongly Agree. A proportional‐odds logit regression was used to assess whether there was a significant change in attitudes between the two survey waves (see Supporting Information S1: Table A2; Hutcheson and Moutinho 2008). This single‐item outcome measure was chosen to prioritise the interpretability of our results, rather than constructing a more complex variable from multiple items. However, recognising that this decision increased the risk of measurement error (M. S. Allen et al. 2022), a sensitivity analysis was performed. This included: (1) the use of an alternative item relating to commonalities: ‘*To what extent do you agree with the statement: I have things in common with Muslim people*’, and (2) the construction of an outcome variable that took a more liberal definition of change (see paragraph below). Following best practice, any divergence in results from the sensitivity analysis are commented on within the findings (Gelman et al. 2020).

An outcome variable was constructed to capture how students' attitudes change over time. We constructed a relatively conservative measure of change, to reduce the risk of overstating the changes that occur during HE, with three values:

- Change Positive:
  o When students moved from a position of disagreement to ‘Neither Agree nor Disagree’ or agreement. Or,
  o Students moved from a position of ‘Neither Agree nor Disagree’ to agreement.
- No Change:
  o When students remained in a position of disagreement, in a position of agreement, or in the position of ‘Neither Agree nor Disagree’.
- Change Negative:
  o When students moved from a position of ‘Neither Agree nor Disagree’ to disagreement. Or,
  o When students moved from a position of agreement to ‘Neither Agree nor Disagree’ or disagreement.

This outcome variable is labelled a ‘conservative’ measure of change, as it considers movement within a position (e.g., from ‘Strongly Agree’ to ‘Agree’) as no change. This approach was adopted to enable us to identify changes in the *type* of attitude (e.g., ‘Agree’ to ‘Disagree’), rather than a change simply in the *level* of agreement (e.g., ‘Strongly Agree’ to ‘Agree’). An alternative outcome variable that took a more ‘liberal’ version of change was also calculated and used within the sensitivity analysis. This liberal variable was constructed as above, with the exception that those moving from ‘Disagree’ to ‘Strongly Disagree’ and from ‘Strongly Agree’ to ‘Agree’ were labelled ‘Change Negative’; and those moving from ‘Strongly Disagree’ to ‘Disagree’ and from ‘Agree’ to ‘Strongly Agree’ were labelled ‘Change Positive’.

After this assessment of whether students' attitudes to Muslims change over time, the article explores a series of hypothesised mechanisms through which different HE experiences may exert an influence on attitudinal change.^3^ Informed by the existing literature on the relevance of provocative encounters (Shaheen et al. 2023), we tested whether students were more likely to change attitudes when they engaged with people from diverse backgrounds. However, our focus here is on ‘proactive encounters’ rather than ‘provocative encounters’; while the two share the sense of engagement with diversity, the former preserves an additional quality of pre‐meditated agency. In other words, we ask whether students have initiated a conversation in the hope of learning about another's beliefs and values. Two other important factors were considered: the students' prior socialising (if they had socialised with a diverse group of people before HE) and HEI diversity (if the HEI has a diverse community of students). The three variables were measured as follows:

- Proactive Encounter, a binary variable (Yes/No), was constructed from the question: *Please indicate whether you have experienced the following since starting university: Initiated a conversation with someone of a different religious or non‐religious perspective to learn about the person's beliefs and values?*
- Prior Socialising, a binary variable (Yes/No), was constructed from the question: *Please indicate whether you participated in any of the following activities before university: Socialised with someone of a different religious or non‐religious perspective?*
- As a proxy for HEI Diversity, an ordered categorical variable was created by dividing the *Sunday Times'* (2023) Diversity Index into quintiles—quintiles were used to mitigate against comparability issues between English, Scottish, Welsh and Irish providers (see Boeren 2024). This index was constructed using data on the percentage of state school students, ethnic minorities, ethnic attainment gaps, white working‐class males, low participation areas, first generation students, disabled and mature students at each HEI.

We initially tested a main effects model involving the three variables above: *Attitudinal Change ∼ Proactive Encounter + Prior Socialising + HEI Diversity* (see Supporting Information S1: Tables A3–A4). Significance was assessed through Type III ANOVA tests for individual parameters, Type II ANOVA tests for overall variables, and polynomial contrast coding was applied to HEI diversity (Hutcheson and Moutinho 2008). Non‐significant variables were removed, and two subsequent models were explored: (1) *Attitudinal Change ∼ Proactive Encounter + Prior Socialising* and (2) Attitudinal Change ∼ Proactive Encounter * Prior Socialising (see Supporting Information S1: Tables A5–A10). Models were analysed using multinomial logit regression as the prior socialising variable violated the assumption of ordinality, making proportional‐odds logit regression inappropriate (Hoffmann 2004).

## 4 Findings

### 4.1 Students' Views of Muslims, and How These Change Over One Year in HE

Our results suggest that most students hold positive attitudes towards Muslims (see Table 1). Broadly speaking, only around 5% of students expressed some level of disagreement with the idea that Muslim people make positive contributions to society, while around two‐in‐three signalled their agreement. Although few students displayed negative attitudes, there were a substantial minority (∼27%) that answered ‘Neither Agree nor Disagree’. This latter response could potentially indicate some scepticism towards the contributions Muslim people make, but equally could reflect a sceptical attitude towards the idea that *anyone* can make a positive contribution to society or a negative stance towards some but not all Muslims (e.g., Muslim immigrants). Hence, while around one‐in‐three did not agree with the statement that Muslim people make positive contributions to society, the low number of students that expressed outright disagreement provides little evidence of the anti‐Muslim attitudes noted by Jones and Unsworth (2024) in our sample of students.

**TABLE 1:** In general, muslim people make positive contributions to society (*n* = 996).

	Frequency		Percentage (%)	
	Wave 1	Wave 2	Wave 1	Wave 2
Strongly disagree	16	21	1.6	2.1
Disagree	33	30	3.3	3.0
Neither agree nor disagree	285	258	28.6	25.9
Agree	424	454	42.6	45.6
Strongly agree	238	233	23.9	23.4

Turning to the question of whether students' attitudes changed over time (see Table 2), we found a large degree of stability; over seven‐in‐ten (70.9%) students maintained the same type of attitude. Of the change that did occur, 15.8% displayed more favourable attitudes and 13.4% reported more negative attitudes towards Muslims. This means that there was a small net positive movement of 2.4% points, over the 12‐month period considered in this analysis.

**TABLE 2:** Changes in attitudes to Muslim people's contributions to society (*n* = 996).

	Frequency	Percentage (%)
Change negative	133	13.4
No change	706	70.9
Change positive	157	15.8

**FIGURE 1:**
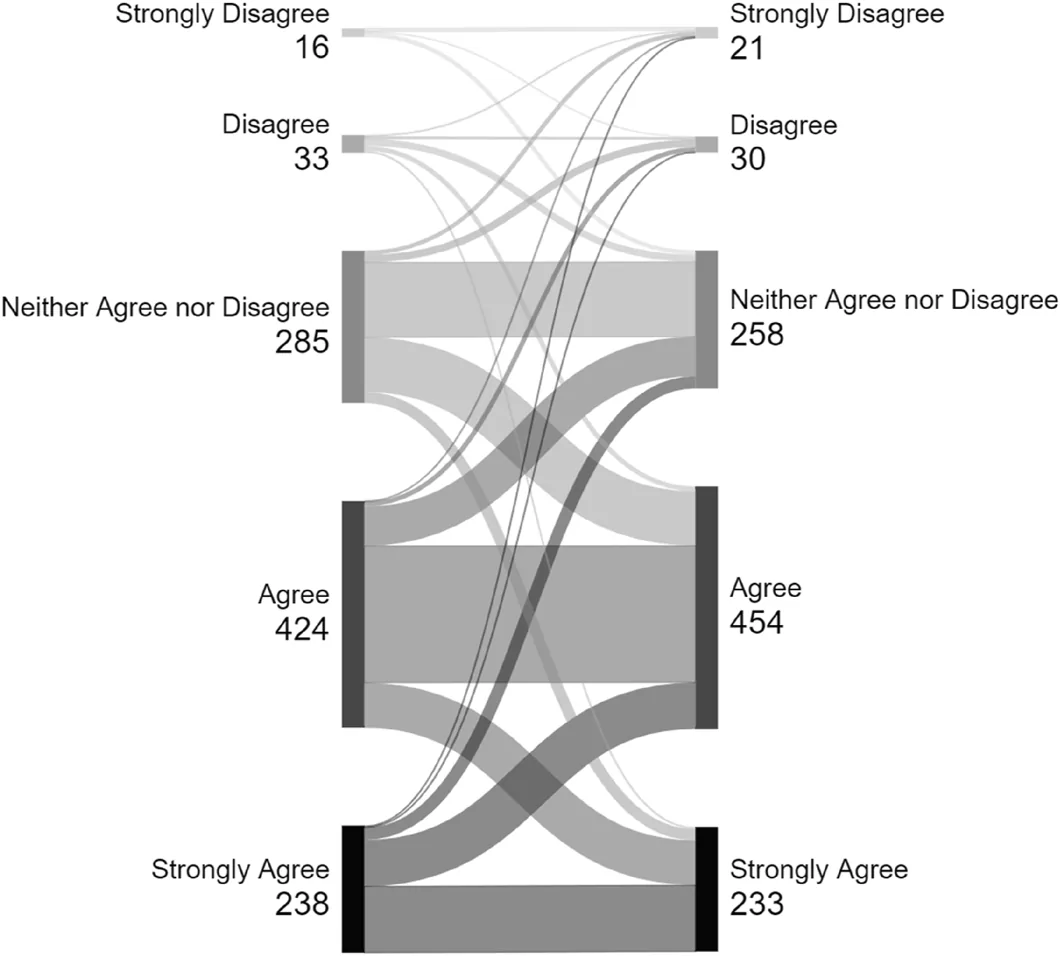
Sankey diagram showing change from Wave 1 (LHS) to Wave 2 (RHS): in general, Muslim people make positive contributions to society (*n* = 996).

A more fine‐grained analysis of this individual level change is shown in the Sankey diagram in Figure a. Again, the relative stability of attitudes is evident, with over one‐in‐two students (53.2%) reporting the exact same answer on the Likert scale in Waves 1 and 2. However, there is a statistically significant difference (*p* < 0.1) between Waves 1 and 2 (see Supporting Information S1: Table A2), with a particular increase in agreement in Wave 2. Figure 1 reveals that most of the positive change in attitudes is a movement from ‘Neither Agree nor Disagree’ (−2.7% points) to ‘Agree’ (+3.0% points). This helps to clarify that the net positive movement of 2.4% points should not be interpreted as a movement of students from a position of disagreement to agreement, but the more complex shift from ‘Neither Agree nor Disagree’ to agree.

It is also important to explore the number of students experiencing substantial changes in attitudes, that is those that shift from a position of disagreement to agreement, or vice versa. Figure 1 suggests that this substantial change of attitude is rare. Only 1.4% (14 students) moved from a position of agreement to disagreement, while 1.3% (13 students) moved in the opposite direction. However, despite only small numbers of students changing their attitudes in this way, we do see that over one‐in‐four of those who reported disagreement in Wave 1 switched to agreement in Wave 2.

### 4.2 How do Students' Attitude Changes to Muslims Relate to Their HE Experiences?

We then investigated whether students' experiences in HE were related to their attitudinal changes. Specifically, we assessed the idea that students would be more likely to change attitudes to Muslims, if HE was the first time they had socialised with someone who held a different worldview, if they initiated a conversation about worldviews during HE, and if they studied in a diverse HE setting.

We began by testing the model *Attitudinal change ∼ Prior Socialising + Proactive Encounter + HEI Diversity Quintile* and found that HEI diversity was not significant (see Supporting Information S1: Tables A3–A4), a finding that was consistent throughout the sensitivity analysis. In response to this result, our subsequent modelling did not include the HEI diversity variable.

**FIGURE 2:**
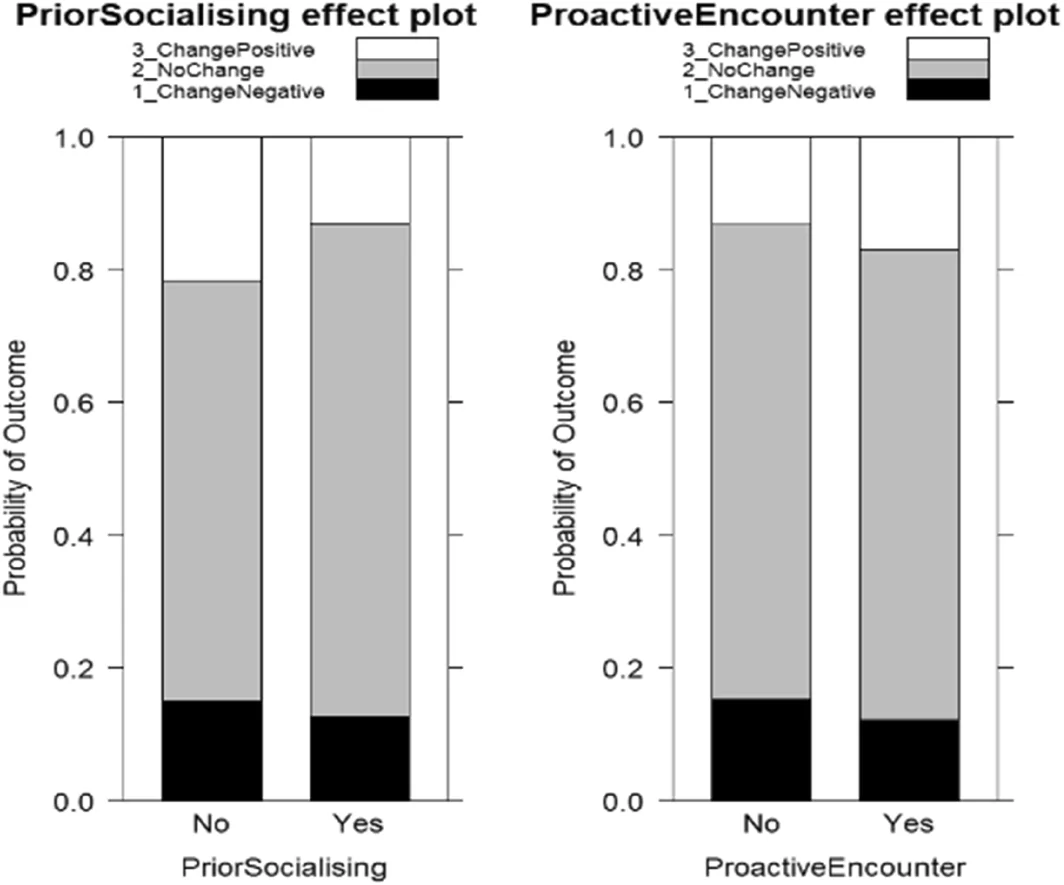
Effect plot for model: attitudinal change ∼ prior socialising + proactive encounter.

Our next model, *Attitudinal change ∼ Prior Socialising + Proactive Encounter,* provided some evidence that prior diverse social experiences and initiating a conversation about worldviews during HE were both related to attitudinal change (see Figure 2). First, prior socialising was significantly associated with attitudinal change (*p* = 0.001): students who had not socialised with someone who held a different worldview before entering HE were more likely to display positive attitudinal change. The model predicted that 22% of those without diverse religious social experiences prior to HE would become more favourable to Muslims, with 63% staying the same, and 15% becoming less favourable. Corresponding values for those who had experienced this prior socialisation were: 13% change positive, 74% stay the same, and 13% change negative.^4^ Therefore, those without diverse prior social experiences were more likely to change attitudes in a positive way, compared with their peers, and this was found to be reasonably robust to changes in the sensitivity analysis.

Second, students who had a proactive encounter, that is they initiated a conversation with someone of a different religious or non‐religious perspective to learn about the person's beliefs and values, were more likely to demonstrate positive attitudinal change towards Muslims (see Figure 2). For those engaged in these encounters, 17% changed positive, 71% stayed the same, and 12% changed negatively; compared with those without these experiences, 13% changed positive; 72% stayed the same, and 15% changed negatively. Framed another way, the odds of changing in a positive rather than negative direction were 64.1% higher for those who initiated at least one conversation about worldviews (*p* = 0.042). This finding was found to be reasonably robust to changes within the sensitivity analysis.

We proceeded to investigate whether there was an interaction between the two variables. In other words, were those without prior diverse socialising who then do initiate a conversation about worldviews particularly likely to change? This was done by testing the model: Attitudinal Change ∼ Prior Socialising * Proactive Encounter.

**FIGURE 3:**
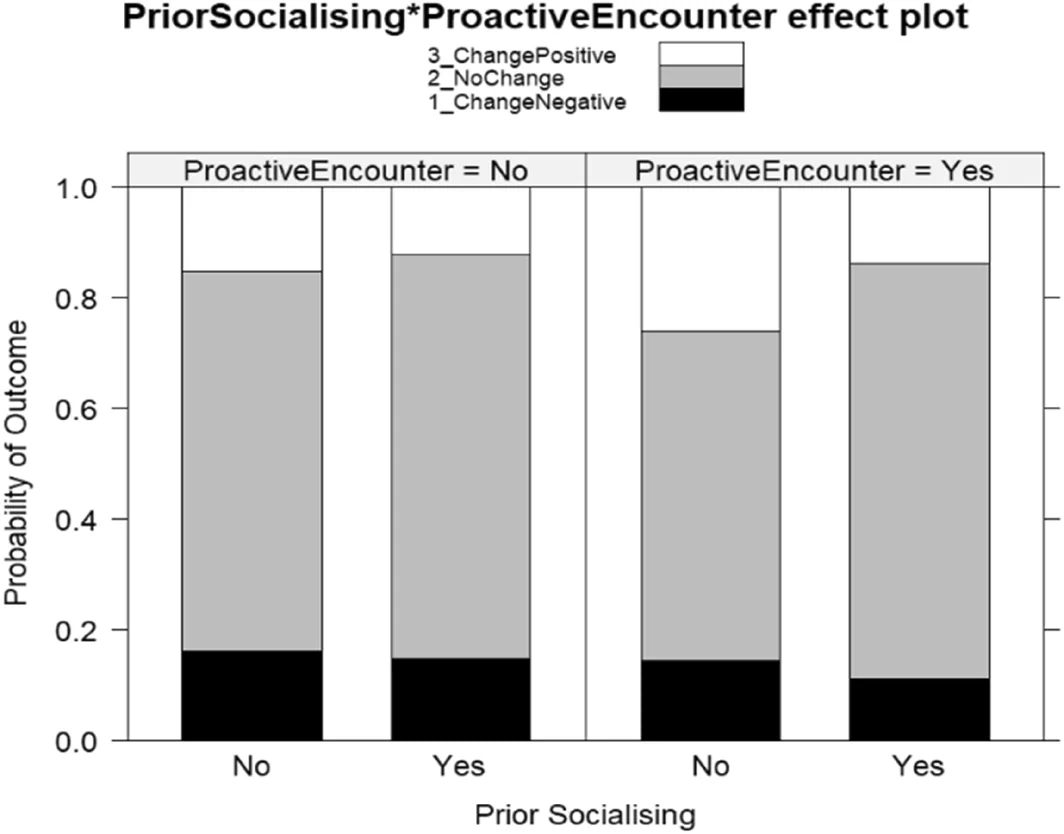
Effect plot for model: attitudinal change ∼ prior socialising * proactive encounter.

Our findings reveal some weak evidence for this interaction (see Figure 3). The model predicts 26% of these students would change attitudes in a positive way, compared with 15% (no prior social, no proactive encounter), 14% (prior social, proactive encounter), and 12% (prior social, no proactive encounter) students with other experiences. Therefore, the model does predict that those without diverse prior socialisation who then initiate a conversation about worldviews were more likely to change positively than not change, compared with their peers, although this result was not quite statistically significant (*p* = 0.136). The contribution‐liberal measure did reveal a significant interaction (*p* = 0.0214), however the commonalities‐conservative and commonalities‐liberal measures provided non‐significant interaction. Overall, we treat this as weak evidence of an interaction.

## 5 Discussion and Conclusion

On our first research question, how students' views of Muslims change during their time at university, we found a positive tendency. Our longitudinal analysis, while restricted to a 12‐month period, demonstrates a small net positive change in those agreeing Muslim people make a positive contribution to society, with 15.8% reporting more favourable attitudes and 13.4% less favourable attitudes in Wave 2, a 2.4% point change. Our data do not include a comparative dimension with non‐university students of the same age group, and so our claims about university itself being a critical factor need to be evaluated with this in mind. However, this is the first UK study of student perspectives on religion to include a longitudinal dimension, and this itself provides a strong evidence base in support of universities fostering what Rockenbach et al. (2020) call ‘appreciative attitudes’ towards Muslims.

It is worth noting that the net positive change identified in our study, while statistically significant, does not encompass large proportions of the student population. A relationship of stability was far more common across the two data points; students were much more likely to start with a positive view of Muslims and retain this. Moreover, the snapshot measures of attitudes in both surveys indicate a much higher proportion affirming positive attitudes towards Muslims than any of the levels cited in available studies of the general population, reinforcing the picture of universities as characterised by a favourable to Muslims orientation that is atypical for the UK context. However, these findings should not be interpreted as evidence that UK universities should be unconcerned about religious discrimination and prejudice on campus. While our results suggest widespread attitudes of acceptance and appreciation, this can occur alongside some significant hostility, as evidenced elsewhere in the literature (Aune, Fryer, et al. 2025; Arday and Mirza 2018; Sheldon 2016; Weller et al. 2011).

The next part of our analysis, related to our second research question, concerns the mechanisms that drive this overall pattern. Interestingly, we did not find evidence that the diversity of a university's student population makes a significant difference. Our proxy for ‘diversity’ was the *Sunday Times* Diversity Index, which aggregates statistical measures of ethnicity, disability, age, prior schooling and socioeconomic background. It is possible that this measure, in collating various sub‐measures, masks important diversity variables that would, if isolated in a cross‐sector analysis, expose a significant influence on student attitudes. Alternatively, it is possible that all universities were sufficiently diverse to enable students to engage in conversations with people with different worldviews, and that variant levels of campus diversity had little discernible impact. A specific measure of religious diversity for each university is not available, and future research will need to explore methods of gauging this in order to pursue a more nuanced analysis.

More significant, according to our analysis, are *proactive encounters* in which students initiate a conversation with someone of a different religious or non‐religious perspective to learn more about their beliefs and values. Students who said they had done this since starting university were significantly more likely to have attitudes to Muslims that changed in a positive direction than those who had not initiated this kind of conversation. In this sense, proactive encounters function similarly to the ‘provocative encounters’ discussed in the US literature (Mayhew et al. 2023), although it is important not to over‐interpret attitudinal change identified here as a more thoroughgoing re‐evaluation of prior assumptions. For example, it is unclear whether the most significant part of proactive encounters is the social *exposure* to religious diversity, the *initiation of a conversation* focused on religious diversity, or the consequent *learning experience* about religious diversity. Further research will need to disentangle these dynamics in order to ascertain their relative significance. What remains clear is that, if positive attitudes towards those of different religious identities to oneself are encouraged by the experience of provocative, challenging encounters that contest pre‐existing assumptions, there is also an important role to be played by the initiation of such encounters among students themselves. In other words, for universities to harness their potential for enabling religious inclusivity, some thought should be given not just to the demographics of student mixing, but to encouraging students to exercise their agency in engaging with those who are different from themselves.

Finally, we have the data on prior religious interaction, or more specifically, whether students had, before coming to university, socialised with someone of a different religious or non‐religious perspective. Those who had not were more likely to develop positive attitudes towards Muslims, suggesting that the impact of universities varies for different students, depending on their pre‐university experience. Then there is the finding, albeit one that received only weak support from the sensitivity analysis, that those with lower levels of prior religious interaction, but who nevertheless initiated a conversation at university with someone from a different religious perspective, were *even more likely* to develop positive attitudes towards Muslims. This suggests the initiation of such a conversation is especially consequential among those making a more significant cultural step; in other words, such proactive encounters make the most difference among those less used to such cross‐religious engagement. This is echoed in the qualitative study of women in US colleges conducted by Yilmaz, who found that interfaith interactions at university fostered ‘transformative experiences’, especially among those who had had limited encounters with religious diversity before college (Yılmaz 2024, 256). Using the language of widening participation, this underlines the potential power of the university experience in instilling multi‐faith positivity among those relatively unfamiliar with experiences of religious diversity.

### 5.1 Implications

Our findings illuminate the social mechanisms that inform the influence the university experience has on students' attitudes, hence contributing to sociological debates about *how* universities shape positive attitudes towards social and religious minorities (Sobolewska and Ford 2020; Scott 2022; Fryer 2023). As outlined above, especially noteworthy are the initiation of inter‐worldview conversations and the extent to which this marks a heightened experience of diversity compared to pre‐university life. Given we have found a majority of students tend to display positive attitudes towards Muslims, and that there is a large degree of attitudinal stability over time, this suggests the importance of factors that pre‐date university, including the selection bias favouring those families with pre‐existing liberal worldviews, charted by other studies (Simon 2022). However, along with Scott (2022) we do find evidence of HE‐specific mechanisms, including that pro‐active engagement with people holding different worldviews tended to lead to students holding more positive attitudes towards Muslims. This echoes existing evidence on the importance of peer‐effects (Strother et al. 2020), suggesting active engagement is important, although further qualitative research is needed to unpack whether these engagements allow students to simply identify and adopt community norms or enable a deeper re‐evaluation of their values.

These conclusions also speak to policy debates about how universities might properly respond to on‐campus religious diversity, especially in light of legislation concerning equality and freedom of speech. The UK's Equality Act (2010) names ‘religion or belief’ as one of its nine ‘protected characteristics’, and universities must have due regard to the elimination of unlawful discrimination, the advancement of equality of opportunity, and the fostering of ‘good relations between people who share and people who do not share a relevant protected characteristic’ (UK Government 2023). Universities therefore have a legal duty to attend to discrimination against Muslims (students and staff), and a vested interest in understanding how they, as institutions, both promote and contest such discrimination, and how they might foster good relations between Muslims and non‐Muslims. At the same time, the recently introduced Higher Education (Freedom of Speech) Act (2023) imposes duties on universities to protect the right of individuals to express controversial views, including perspectives on religion that some might find offensive. As the HE sector negotiates this tricky terrain, universities could learn much from research that illuminates how their institutional environments accommodate or inhibit the expression of religious—including Muslim—identities, and the views students hold towards those different from themselves. The above analysis begins to shed light on these institutional mechanisms, most notably how ‘proactive encounters’ are associated with positive attitudinal change. It is the responsibility of university leaders to facilitate such encounters while guarding against their slippage into discriminatory and/or coercive interaction (Aune, Peacock, et al. 2025). This is a challenging balance to strike, but one that, we would argue, is essential to the capacity of UK universities in fostering healthy educational environments for those affirming a range of religious and non‐religious worldviews.

## Funding

The authors disclosed receipt of the following financial support for the research, authorship, and/or publication of this article: This work was supported by Porticus, The Spalding Trust and Durham University.

## Conflicts of Interest

The authors declare no conflicts of interest.

## Supporting information


Supporting Information S1

## Data Availability

Research data are not shared.

## Endnotes

^1^
YouthSight were asked to construct a sample that approximated the overall UK student population according to HESA data, based on gender and ethnicity. YouthSight were also asked to sample equally across year of study.

^2^
IDEALS UK weights were created using generalized ranking applied to gender, ethnicity, age, university type and level of study, which resulted in some respondents being assigned large weights. Our analysis was found to be sensitive to the deletion of these high weight cases, and therefore, we decided not to use the weights. This avoids a small number of participants skewing our results but does maintain the sample bias (see Supporting Information S1: Table A1).

^3^
This paper explores some, but not all, mechanisms by which HE could influence students' attitudes. For example, our analysis did not assess the impact of specific diversity interventions organised by universities.

^4^
Figure 2 suggests those without diverse prior social experiences were also more likely to change negatively, compared with their peers. However, this result was not found to be robust within the sensitivity analysis; no other significant results for this increased likelihood of negative change.
