# Supplementary material for: How Does Higher Education Influence Attitudes Towards Muslims? Examining Mechanisms That Reduce Prejudice Within UK Universities
Source: Br J Sociol. 2026 Apr 7;77(4):690–700. doi: 10.1111/1468-4446.70113 (PMC13536525; doi:10.1111/1468-4446.70113)
Supplement: Supplementary file 1 [file BJOS-77-690-s001.docx]

Appendix

# Sample

Table AI: Comparison of longitudinal sample and HESA data from 2020-21

|  | HESA (2020-21) | Longitudinal Sample |
| --- | --- | --- |
| HEI Type | Russell Group: 26%  Non-Russell Group: 74% | Russell Group: 42%  Non-Russell Group: 58% |
| Domicile | UK: 78%  International: 22% | UK: 83%  International: 17% |
| Mode of study | Full time: 82%  Part time: 18% | Full time: 98%  Part time: 2% |
| Level of study | Undergrad: 75%  Postgrad: 25% | Undergrad: 89%  Postgrad: 11% |
| Subject area | Humanities & Social Science: 55%  STEM & Medicine: 45% | Humanities & Social Science: 40%  STEM & Medicine: 60% |
| Gender identity | Female: 57%  Male: 43%  Other: <1% | Female: 69%  Male: 29%  Other: 2% |
| Age | 18-20: 41%  21-24: 30%  25+: 30% | 18-20: 69%  21-24: 21%  25+: 9% |
| Ethnicity  *UK domiciled students | Asian: 12%  Black: 8%  Mixed: 4%  Other: 2%  White: 73% | Asian: 18%  Black: 2%  Mixed: 5%  Other: 2%  White: 73% |
| Religion | Non-religious: 48%  Buddhist: 1%  Christian: 32%  Hindu: 4%  Jewish: <1%  Muslim: 11%  Sikh: 1%  Other: 3% | Non-religious: 53%  Buddhist: 2%  Christian: 25%  Hindu: 4%  Jewish: 1%  Muslim: 12%  Sikh: 1%  Other: 3% |

HESA Data from Higher Education Student Statistics: UK, 2021/22 statistical bulletin. This was limited to those on First Undergraduate Degrees, and Postgraduate Taught. Also, ‘Not Known’ was excluded from HESA data.

# Statistical Output

Table AII: Analysis of Deviance Table (Type II test) for model: Attitude ~ Time + ID

|  | LR Chisq | Df | Pr(>Chisq) |
| --- | --- | --- | --- |
| Time | 3.04 | 1 | 0.08123 · |
| ID | 1956.63 | 995 | < 2e-16 *** |

Signifiance codes: 0 '***' 0.001 '**' 0.01 '*' 0.05 '.' 0.1 ' ' 1

Where: Attitude is view on Muslim people’s contribution to society (Disagree; Neither Agree nor Disagree; Agree); Time is Wave 1/Wave 2; and ID is unique identifier for each person.

Table AIII: Output for z test of coefficients for model: Attitudinal Change ~ Prior Socialising + Proactive Encounter + HEI diversity

|  | Estimate | Std. Error | z value | Pr(>\|z\|) |
| --- | --- | --- | --- | --- |
| 2_NoChange:(Intercept) | 1.362821 | 0.213199 | 6.3923 | 1.63e-10*** |
| 2_NoChange:PriorSocial[T.Yes] | 0.296755 | 0.213966 | 1.3869 | 0.16546 |
| 2_NoChange:ProactEncount [T.Yes] | 0.17393 | 0.197638 | 0.88 | 0.37883 |
| 2_NoChange:HEIDiversity.L | -0.05066 | 0.246582 | -0.2055 | 0.83721 |
| 2_NoChange:HEIDiversity.Q | -0.11953 | 0.238397 | -0.5014 | 0.6161 |
| 2_NoChange:HEIDiversity.C | -0.42457 | 0.254998 | -1.665 | 0.09592 · |
| 2_NoChange:HEIDiversity^4 | -0.0004 | 0.238258 | -0.0017 | 0.99865 |
| 3_ChangePositive:(Intercept) | 0.015914 | 0.265696 | 0.0599 | 0.95224 |
| 3_ChangePositive:PriorSocial[T.Yes] | -0.39302 | 0.2598 | -1.5128 | 0.13034 |
| 3_ChangePositive: ProactEncount[T.Yes] | 0.495829 | 0.252644 | 1.9626 | 0.0497 * |
| 3_ChangePositive:HEIDiversity.L | -0.467 | 0.330374 | -1.4135 | 0.1575 |
| 3_ChangePositive:HEIDiversity.Q | -0.24383 | 0.314619 | -0.775 | 0.43835 |
| 3_ChangePositive:HEIDiversity.C | -0.08253 | 0.325666 | -0.2534 | 0.79995 |
| 3_ChangePositive:HEIDiversity.^4 | -0.14885718 | 0.30048442 | -0.4954 | 0.62032 |

Signifiance codes: 0 '***' 0.001 '**' 0.01 '*' 0.05 '.' 0.1 ' ' 1

Table AIV: Analysis of Deviance Table (Type II tests) for model: Attitudinal Change ~ Prior Socialising + Proactive Encounter + HEI diversity

|  | LR Chisq | Df | Pr(>Chisq) |
| --- | --- | --- | --- |
| PriorSocial | 12.8139 | 2 | 0.00165 ** |
| ProactEncount | 4.1989 | 2 | 0.12252 |
| HEI Diversity | 9.035 | 8 | 0.33935 |

Signifiance codes: 0 '***' 0.001 '**' 0.01 '*' 0.05 '.' 0.1 ' ' 1

Table AV: Output for z test of coefficients for model: Attitudinal Change ~ Prior Socialising + Proactive Encounter

|  | Estimate | Std. Error | z value | Pr(>\|z\|) |
| --- | --- | --- | --- | --- |
| 1_ChangeNegative:(Intercept) | -1.64356 | 0.15436 | -10.6474 | < 2.2e-16 *** |
| 1_ChangeNegative:PriorSocial[T.No] | 0.34203 | 0.2048 | 1.6701 | 0.094901 · |
| 1_ChangeNegative:ProactEncount [T.Yes] | -0.21665 | 0.19084 | -1.1353 | 0.256263 |
| 3_ChangePositive:(Intercept) | -1.89535 | 0.16489 | -11.4944 | < 2.2e-16 *** |
| 3_ChangePositive:PriorSocial[T.No] | 0.66209 | 0.18461 | 3.5864 | 0.000335 *** |
| 3_ChangePositive:ProactEncount [T.Yes] | 0.27855 | 0.18738 | 1.4866 | 0.137126 |

Signifiance codes: 0 '***' 0.001 '**' 0.01 '*' 0.05 '.' 0.1 ' ' 1

Table AVI: Odds ratios for model: Attitudinal Change ~ Prior Socialising + Proactive Encounter

|  | (Intercept) | PriorSocial[T.No] | ProactEncount [T.Yes] |
| --- | --- | --- | --- |
| 1_ChangeNegative | 0.193291 | 1.407806 | 0.805212 |
| 3_ChangePositive | 0.150266 | 1.938837 | 1.321214 |

Table AVII: Analysis of Deviance Table (Type II tests) for model: Attitudinal Change ~ Prior Socialising + Proactive Encounter

|  | LR Chisq | Df | Pr(>Chisq) |
| --- | --- | --- | --- |
| PriorSocial | 13.5496 | 2 | 0.001142 ** |
| ProactEncount | 4.2469 | 2 | 0.119621 |

Signifiance codes: 0 '***' 0.001 '**' 0.01 '*' 0.05 '.' 0.1 ' ' 1

Table AVIII: Output for z test of coefficients for model: Attitudinal Change ~ Prior Socialising * Proactive Encounter

|  | Estimate | Std. Error | z value | Pr(>\|z\|) |
| --- | --- | --- | --- | --- |
| 1_ChangeNegative:(Intercept) | -1.59071 | 0.167321 | -9.507 | <2e-16 *** |
| 1_ChangeNegative:PriorSocial[T.No] | 0.147317 | 0.317342 | 0.4642 | 0.6425 |
| 1_ChangeNegative:ProactEncount[T.Yes] | -0.31173 | 0.229015 | -1.3612 | 0.1735 |
| 1_ChangeNegative:PriorSocial[T.No]:ProactEncount[T.Yes] | 0.33102 | 0.415789 | 0.7961 | 0.426 |
| 3_ChangePositive:(Intercept) | -1.76833 | 0.180324 | -9.8064 | <2e-16 *** |
| 3_ChangePositive:PriorSocial[T.No] | 0.26425 | 0.330009 | 0.8007 | 0.4233 |
| 3_ChangePositive:ProactEncount [T.Yes] | 0.076236 | 0.23007 | 0.3314 | 0.7404 |
| 3_ChangePositive:PriorSocial[T.No]:ProactEncount [T.Yes] | 0.595836 | 0.399822 | 1.4903 | 0.1362 |

Signifiance codes: 0 '***' 0.001 '**' 0.01 '*' 0.05 '.' 0.1 ' ' 1

Table AIX: Odds ratios for model: Attitudinal Change ~ Prior Socialising * Proactive Encounter

|  | (Intercept) | PriorSocial[T.No] | ProactEncount[T.Yes] | PriorSocial[T.No]:ProactEncount [T.Yes] |
| --- | --- | --- | --- | --- |
| 1_ChangeNegative | 0.20378 | 1.158722 | 0.732182 | 1.392387 |
| 3_ChangePositive | 0.170618 | 1.302454 | 1.079217 | 1.814546 |

Table AX: Analysis of Deviance Table (Type II tests) for model: Attitudinal Change ~ Prior Socialising * Proactive Encounter

|  | LR Chisq | Df | Pr(>Chisq) |
| --- | --- | --- | --- |
| PriorSocial | 13.5496 | 2 | 0.001142 ** |
| ProactEncount | 4.2469 | 2 | 0.119621 |
| PriorSocial:ProactEncount | 2.5335 | 2 | 0.281742 |

Signifiance codes: 0 '***' 0.001 '**' 0.01 '*' 0.05 '.' 0.1 ' ' 1

# Sensitivity Analysis

The sensitivity analysis for Table I, utilising the alternative outcome of Muslim commonalities (*I have things in common with Muslim people)*, found:

- Wave 1: Strongly Disagree 3.7%; Disagree 9.1%; Neither agree nor disagree 29.9%; Agree 40.2%; Strongly Agree 17.1%;
- Wave 2: Strongly Disagree 2.8%; Disagree 7.6%; Neither agree nor disagree 26.0%; Agree 44.0%; Strongly Agree 19.6%.

The sensitivity analysis for Table II found net-positive changes:

- Contribution-liberal measure: 24.4% change positive; 53.2% same, 22.4% change negative;
- Commonalities-conservative measure: 20.9% change positive, 66.5% same, and 12.7% change negative;
- Commonalities-liberal measure: 29.4% change positive, 51.6% same, and 19.0% change negative.

The sensitivity analysis for *Attitudinal Change ~ Prior Socialising* + *Proactive Encounter + HEI Diversity* found:

- Contribution-liberal measure: *HEI Diversity* was not statistically significant (p=0.76);
- Commonalities-conservative measure: *HEI Diversity* was not statistically significant (p=0.42);
- Commonalities-liberal measure: *HEI Diversity* was not statistically significant (p=0.43).

The sensitivity analysis for *Attitudinal Change ~ Prior Socialising* + *Proactive Encounter* (see Figure II) found:

- Reasonably robust evidence that the odds of changing positive rather than not changing were higher for those who had not previously socialised with someone who held a different worldview:
  - Contribution-liberal measure: the odds of changing positive rather than not changing were 61.6% higher for those who had not socialised with someone who held a different worldview (p=0.004);
  - Commonalities-conservative measure: odds of changing positive rather than staying the same were 31.1% higher (p=0.12), which was almost significant at the p<0.1 level;
  - Commonalities-liberal measure: the odds of changing positive rather than staying the same were 13.6% higher, but this was not statistically significant (p=0.43).
- No evidence that the odds of changing negative rather than not changing were higher for those who had not previously socialised with someone who held a different worldview:
  - Contribution-liberal measure: a non-significant result was found (p=0.64)
  - Commonalities-conservative measure: a non-significant result was found (p= 0.25)
  - Commonalities-liberal measure: a non-significant result was found (p= 0.96)
- Reasonably robust evidence that the odds of changing positive rather than changing negative were higher for those that had a proactive encounter:
  - Contribution-liberal measure: the odds of changing positive rather than changing negative were 49.2% higher for those who initiated a conversation about worldviews (p=0.036);
  - Commonalities-conservative measure: the odds of changing positive rather than changing negative were around 40.1% (p=0.144);
  - Commonalities-liberal measure: the odds of changing positive rather than changing negative were around 33.0% (p=0.138).
  - Note, an alternative comparison between changing positive and not changing yielded similar results for those that had a proactive encounter. Odds of changing positive versus not changing were: (1) contribution-conservative: 32.1% higher (p=0.14); (2) contribution-liberal: 24.3% higher (p=0.18); (3) commonalities-conservative: 20.5% higher (p=0.26); (4) commonalities-liberal: 37.2% higher (p=0.038).

The sensitivity analysis for *Attitudinal Change ~ Prior Socialising* * *Proactive Encounter* (see Figure III) found:

- Contribution-liberal measure: the interaction term was statistically significant (p=0.0214);
- Commonalities-conservative measure: the interaction term was not statistically significant (p=0.533);
- Commonalities-liberal measure: the interaction term was not statistically significant (p=0.604).
